# Differential protein expression in perfusates from metastasized rat livers

**DOI:** 10.1186/1477-5956-11-37

**Published:** 2013-07-29

**Authors:** Yang Zhang, Menglin Li, Lilong Wei, Lisi Zhu, Siqi Hu, Shuzhen Wu, Sucan Ma, Youhe Gao

**Affiliations:** 1 National Key Laboratory of Medical Molecular Biology, Department of Physiology and Pathophysiology, Institute of Basic Medical Sciences, Chinese Academy of Medical Sciences/School of Basic Medicine, Peking Union Medical College, Beijing 100005, China; 2 Department of Neurosurgery, Beijing Tiantan Hospital, Capital Medical University, Beijing, China; 3 Core Instrument Facility, Institute of Basic Medical Sciences, Chinese Academy of Medical Sciences/School of Basic Medicine, Peking Union Medical College, Beijing 100005, China

**Keywords:** Liver perfusate proteome, Biomarker discovery, Liver metastasis, Ywhab

## Abstract

**Background:**

Liver perfusates exhibit theoretical advantages regarding the discovery of disease biomarkers because they contain proteins that readily enter the blood-stream, and perfusion preserves the disease state in its natural context. The purpose of the study is to explore the value of liver perfusate proteome in the biomarker discovery of liver diseases.

**Results:**

In this study, 86 differentially expressed proteins were identified in perfusates from isolated rat livers metastasized by Walker-256 tumor cells. Among these proteins, 27 were predicted to be secreted, and 59 were intracellular or membrane proteins. Most of the secretory proteins (70.4%) were decreased in metastasized liver perfusates. The main canonical ingenuity pathway to which these secretory proteins belonged was acute phase response, which indicated that the liver-associated immune reaction was damaged by the metastasis. In contrast, most of the intracellular or membrane proteins (86.4%) exhibited higher relative abundances in the metastasized liver perfusates. Some of these proteins, including Rpl21, Atic, Eif3s2, Echs1, Eps15 and Ywhab, have previously been reported to be involved in cancer genesis and progression. As a member of the 14-3-3 protein family, Ywhab plays a key role in cellular proliferation and oncogenic transformation and has been reported to be involved in the development of breast cancer. Its abundance was elevated by 3.5-fold in the metastasized perfusates. Validation by Western blotting revealed a 3.7-fold increase in the abundance of this protein in metastasized plasma.

**Conclusions:**

These results show that perfusate proteome can be used as an alternative initial resource for biomarker identification, which ultimately requires validation in serum.

## Background

Biomarkers play a pivotal role in disease screening, diagnosis, therapeutic monitoring and prognosis. Because most biomarkers used in the clinical setting are not satisfactory due to their limited specificity and sensitivity [1], an enormous effort has been made to screen and characterize new disease biomarkers during the past several decades. Recently, advances in proteomic technology, with high-throughput as its characteristics, have presented new possibilities to detect disease biomarkers.

Two strategies are routinely employed in current proteomics-based approaches for biomarker discovery [1]. The first characterizes differential protein expression patterns by comparing serum/plasma from animal models or patients with samples from normal controls. The second compares diseased tissues/cells with their normal counterparts. Despite the unique advantages of the first strategy, the extremely complexity of blood presents a significant challenge for the detection of useful potential biomarkers [1,2]. Disease-specific proteins can be characterized relatively more easily by the second strategy. However, most of these proteins remain within the cell and do not enter the bloodstream, which makes them unavailable for measurement via routine blood assays in a clinical setting.

Perfusates contain secretory proteins, membrane proteins and intracellular proteins released by an isolated perfused organ [3]. Because perfusates pass through the same vascular systems in the organ as does the blood, in theory, perfusate proteins can easily enter into the bloodstream and be detected via routine blood assays. Additionally, the structural and functional integrity of the organ remains relatively intact during perfusion, which preserves the disease state more naturally compared to assays performed at the tissue or cellular level. Therefore, perfusates have the innate advantages in discovery of disease biomarkers. Perfusates from isolated rat hearts during reperfusion were collected after brief periods of ischemia, and some known clinical biomarkers for myocardial ischemia were identified [4].

The isolated perfused rat liver (IPRL) model was first introduced by Claude Bernard in 1855. It is a widely employed physiological model for studying liver functions [5,6]. In our previous work, the IPRL model was combined with mass spectrometry to study the liver secretome at the organ level [3]. In the current study, perfusates were collected from rat livers metastasized by Walker-256 cells [7-9]. Eighty-six differentially expressed perfusate proteins were identified and various important proteins related to liver metastasis were identified, which indicates the value of these perfusates for biomarker discovery. To our knowledge, there has not been a study on differential protein expression in the perfusates from metastasized organs aimed at biomarker discovery.

## Results and discussion

### Establishment of the liver metastasis model

Metastasized cancers can form in the liver after inoculation of the spleen with cancer cells because the liver collects the blood from the spleen. In this study, the model of liver metastasis was produced via inoculation of Walker-256 tumor cells into male SD rat spleens, as described previously [10]. On the sixth day after inoculation, tumor tubercles were observed in the spleen (Additional file 1: Image 1). On the ninth day, metastasized tubercles were detected in the liver under visual or microscopic inspection (Additional file 1: Images 2 and 3).

#### The average weight of inoculated rats was much lower than the controls

**Figure 1:**
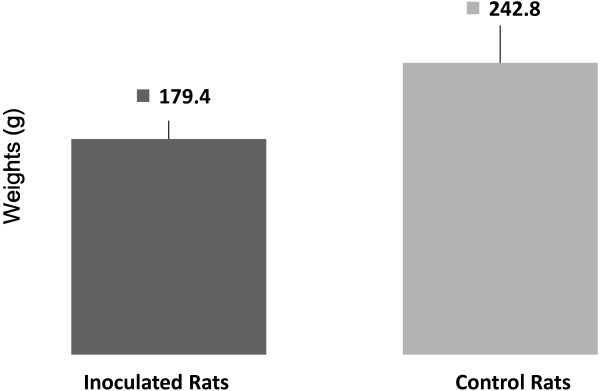
**Rat weight decreased after tumor inoculation.** The average weight of the tumor-inoculated rats (179.4 g) decreased significantly compared to the average weight of control rats (241.8 g). (*p* < 0.05, n = 5).

The average weight of the tumor-inoculated rats was much lower than that of the control rats (Figure 1 and Additional file 1: Table S1). The recorded weights were consistent with those reported in previous studies [8,9], which showed that Walker-256 tumor cells induce a cachectic state.

#### The 11-13th day after inoculation was selected as the time-point for perfusion

**Figure 2:**
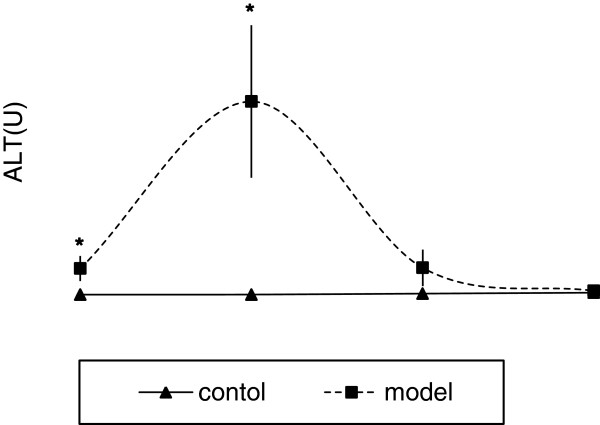
**The degree of liver injury caused by metastasis was recovered on day 12.** The level of alanine aminotransferase (ALT) in the serum was used as a marker of liver injury. The ALT levels increased to the maximum level on the 6th day (Day 6) after inoculation and returned to the control level on the 12th day (Day 12). On the 3rd (Day 3) and 6th day (Day 6), the ALT levels in the model rats were significantly higher than in the control rats, *p* < 0.05(*), (n = 3 for each group).

It was shown by the preliminary study that metastasis induced liver damage and made more intracellular proteins released into the perfusates of metastasized livers. These proteins were more readily identified as differentially expressed proteins by proteomic analysis. In order to discover candidate biomarkers related to liver metastasis, we tried to reduce the effect of metastasis-induced liver damage. So serum alanine aminotransferase (ALT) levels were applied to monitor the damage and to find a time-point when the damage recovered to the normal control level. In Figure 2, ALT levels increased to the maximum level on the 6th day after inoculation and returned to the control level on the 12th day. Therefore, to discover more candidate biomarkers related to metastasis, the period from the 11-13th day was selected as the time-point for perfusion.

### Serum contamination was low in the perfusates

Because immunoglobulins are high-abundance proteins in the serum and are not secreted by the liver [11-14], the amount of immunoglobulins in perfusates can reflect the level of serum contamination [3]. Therefore, IgG was chosen as an indicator of serum contamination in the perfusates. The IgG level, as shown in Additional file 1: Figure S1, indicated that serum contamination was at a very low level.

### The perfusates showed a different protein composition compared to liver cytosol extracts

**Figure 3:**
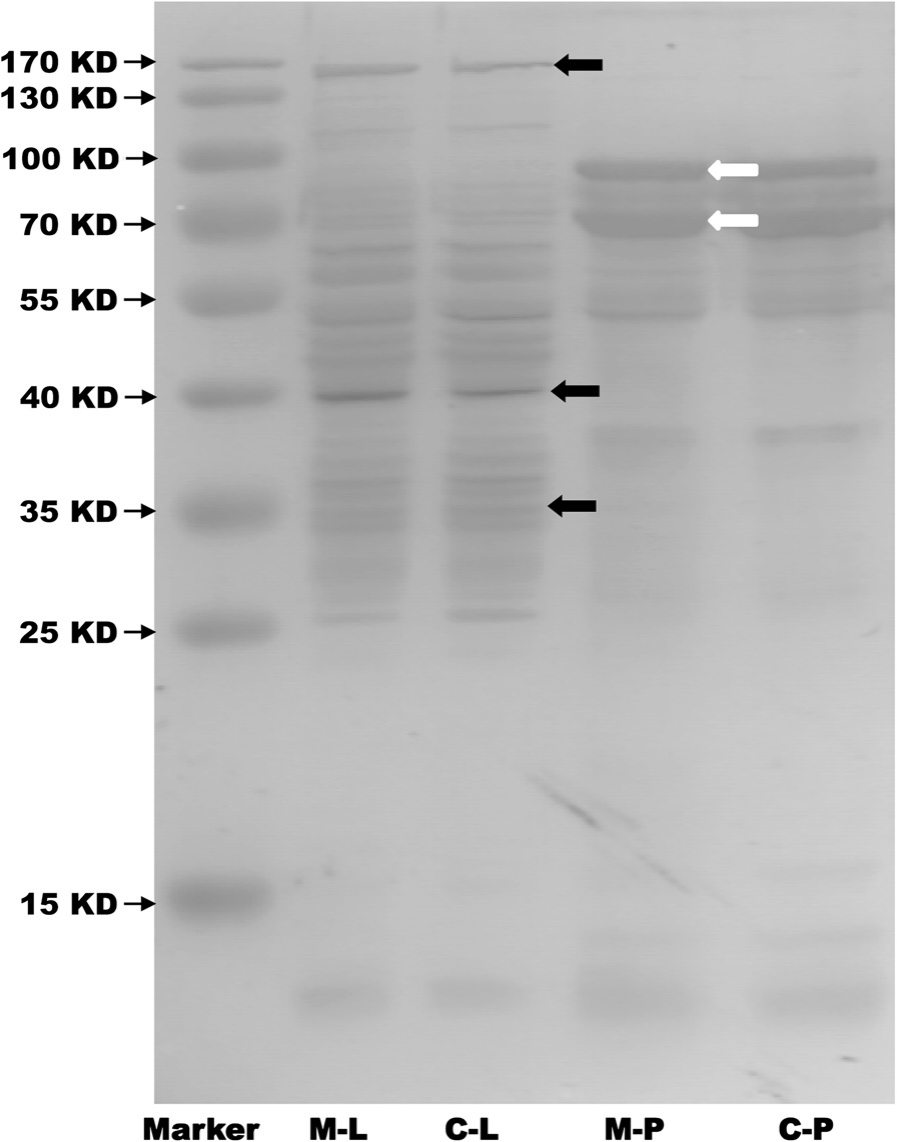
**The perfusates presented a different protein composition compared to liver cytosol extracts.** Eight micrograms of protein from the perfusate and cytosol mixtures^a^ from both the model and control rats was loaded onto the gel, and the gel was subsequently silver-stained. Substantial differences were detected in the perfusate and cytosol. Many high-abundance cytosolic proteins (black arrows) were not observed in the perfusates, whereas some proteins were enriched in perfusates (white arrows). M-L: Liver cytosol mixture from the model rats. C-L: Liver cytosol mixture from the control rats. M-P: Perfusate mixture from the model rats. C-P: Perfusate mixture from the control rats. a. The cytosol mixture was prepared according to our previous work [3]. Briefly, the perfused rat livers were snap-frozen with liquid nitrogen, and then ground into a powder with a pestle. The liver tissue powder was suspended in 40 mM Tris buffer on ice, followed by centrifugation at 12000 g for 30 minutes at 4°C. The supernatants were collected as liver cytosol extracts. The cytosol mixture was prepared by pooling liver cytosolic extracts from five model or control rats.

Figure 3 shows that the SDS-PAGE patterns were significantly different between the perfusate and liver cytosolic extract. Many high-abundance cytosolic proteins (black arrows) were not present in the perfusates, whereas some proteins were enriched in perfusates (white arrows).

### Proteomic profiling of the perfusates

An online SCX-RPLC-MS/MS analysis was performed to identify the proteins in the perfusates from the model and control rats. Five technical replicate analyses were conducted for each pooled perfusate sample. Additional file 1: Table S2 lists proteins numbers and total spectral counts identified in each replicate analysis, with probabilities >0.9, as calculated by ProteinProphet. The average numbers of proteins identified in the model and control perfusates mixture were 771 and 674, respectively. The average of the total spectral counts identified in the model perfusate mixture was 16,222, which was slightly less than the 19,241 total spectral counts identified in the control perfusate mixture. Considering this inconsistency, spectral count weights were used to estimate the relative protein abundances in the perfusates.

### Differential protein expression in the perfusates

The SCW was used to estimate the relative protein abundances in the samples. The difference of each protein’s SCW between the model and control rats was analyzed for statistical significance by Student’s *t*-test. A *p*-value <0.01 was set as a cut-off. To increase the credibility of protein identification and quantification, three strict criteria were used to select differentially expressed proteins (see details in the Experimental Procedures). A total of 59 proteins showed an increased relative abundance, and 27 proteins presented a decreased relative abundance in the model perfusates (Additional file 2: Table S3).

SignalP software is routinely used to predict secretory proteins [15]. SignalP can predict the N-terminal signal peptide contained in classically secreted proteins. Among the 86 differentially expressed proteins, 27 (31.4%) were predicted to be secreted, and 59 (68.6%) were considered non-secretory (intracellular or membrane proteins).

### Functional analysis of the differentially expressed proteins associated with cancer metastasis

Functional analysis of these differentially expressed proteins was performed by the Ingenuity Pathway Analysis (IPA) tool (http://www.ingenuity.com/, Ingenuity Systems, Redwood City, CA, USA). This analysis was focused on the pathways and disease mechanisms in which the proteins are expected to be involved. Secretory and non-secretory proteins were analyzed respectively.

#### Liver-associated immune function was suppressed by metastasis

**Figure 4:**
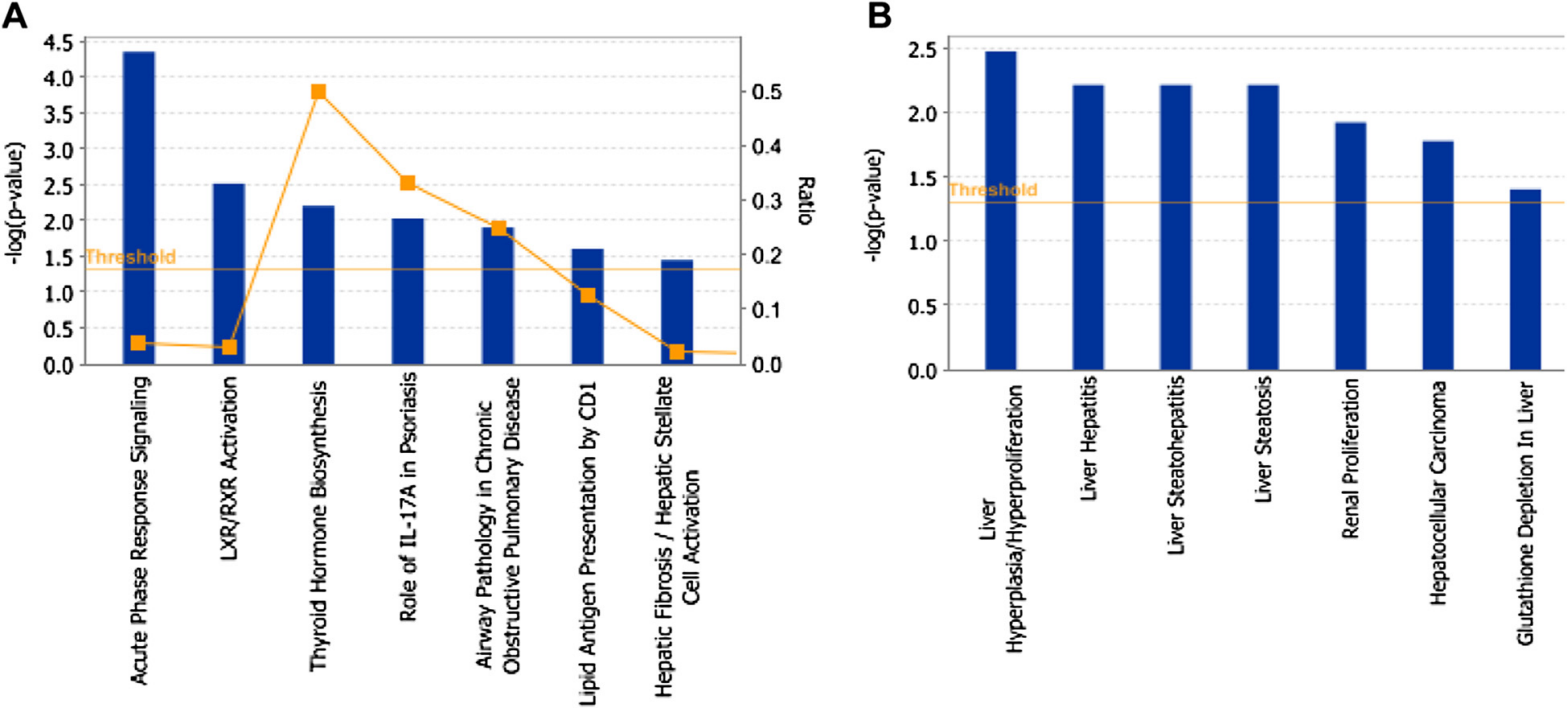
**Functional analysis of the differentially expressed proteins in perfusates. A**. the Ingenuity Canonical Pathway in which the greatest number of secretory proteins participated was acute phase response signaling. **B**. the main diseases in which the non-secretory proteins participated were liver hyperplasia/hyper-proliferation, liver hepatitis, liver steatohepatitis, liver steatosis and hepatocellular carcinoma.

As shown in Figure 4A, the Ingenuity Canonical Pathway in which the greatest number of secretory proteins participated was acute phase response signaling. The acute phase response is a well-known liver function that plays an important role in immune defense. The perfusate proteins involved in this pathway included TTR, MBL2, FN1, CRP and SERPINF1. Most of these proteins showed a decreased relative abundance in metastasized perfusates (Additional file 2: Table S3), which indicated that the liver-associated immune function was damaged by the metastasis or the state of cancer cachexia.

#### Liver cancer-related molecules were found in non-secretory perfusate proteins

The main diseases in which the non-secretory proteins participated were liver hyperplasia/hyper-proliferation, liver hepatitis, liver steatohepatitis, liver steatosis and hepatocellular carcinoma (Figure 4B). These proteins included PAK1, SOD1, BZW1, IQGAP1, GSTO1, MAT1A and FASN. Certain cancer-related pathways were over-represented, such as the PI3, Ras, EGF, FGF and angiogenesis pathways (data not shown). The proteins involved in these pathways were PAK1, Grb2, Ywhab and Ppp2r1a. Most of these proteins showed a higher relative abundance in cancer perfusates and may be involved in metastasis.

#### Certain differentially expressed perfusate proteins have been reported to be associated with metastasis

Some proteins, exhibiting an increased relative abundance in the model perfusate, have been already reported to be associated with cancer metastasis, such as Rpl21 [16], Emilin1 [17], Eif3s2 [18,19], Echs1 [20] and CtsD [21-24]. The relative abundance of CtsD was 5.6-fold elevated in the perfusates from the livers metastasized by Walker-256 tumor cells. CtsD is a lysosomal enzyme, which was reported to be secreted by the breast cancer cells [21,22]. It was also involved in the metastasis of breast cancer and was an independent prognostic biomarker for the cancer [23,24].

### Validation of differentially expressed perfusate proteins via Western blotting using plasma

Three proteins were chosen for Western blot analysis: Beta-2-microglobulin (B2-M), Fibroblast growth factor 21 (FGF 21) and 14-3-3 protein α/β (Ywhab). The plasma for the validation studies was collected from another 8 pairs of model and control rats. Each rat’s plasma was collected at three time-points: on days 0, 6 and 12 (before surgical inoculation and on the sixth and twelfth days after inoculation, respectively). An equal volume of plasma from each rat was loaded onto a gel and analyzed via Western blotting.

**Figure 5:**
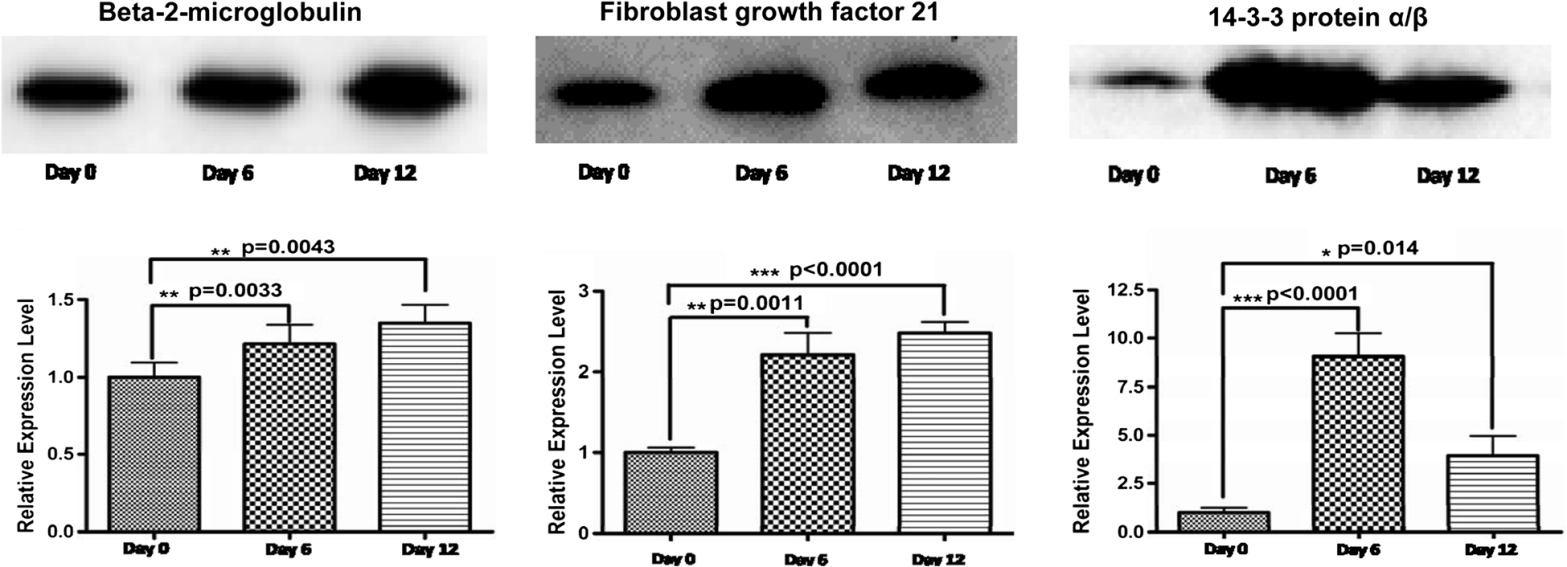
**Validation of differentially expressed perfusate proteins in plasma.** Beta-2-microglobulin (B2-M), Fibroblast growth factor 21 (FGF 21) and 14-3-3 protein α/β (Ywhab) were chosen for validation by Western blotting. The plasma was collected at three time-points from each model rat: on days 0, 6 and 12 (before the surgical inoculation and on the sixth and twelfth day after inoculation, respectively). Equal volumes of plasma were loaded into a gel and analyzed by Western blotting. The change direction in the relative abundance of 14-3-3 protein α/β in the perfusates was the same as in the plasma, whereas the change direction in the relative abundance of the B2-M and FGF 21 proteins was opposite to that in the plasma.

All three proteins were detected in plasma by Western blotting, as shown in Figure 5. Ywhab increased 3.7-fold in the model plasma, consistent with the results from the model perfusates (3.5-fold increased). As a member of the 14-3-3 protein family, Ywhab plays a key role in cellular proliferation and oncogenic transformation [25]. This result indicated that Ywhab may participate in liver metastasis and could be a potential biomarker for this disease.

However, the change direction of B2-M and FGF 21 in the model plasma was opposite to that in the model perfusates (Figure 5 and Additional file 2: Table S3): the relative abundance of these proteins was decreased in perfusates, whereas it was increased in plasma. It is likely that these proteins are not liver-specific proteins and can be secreted by other organs [26,27], which may mask their decreased abundance in the liver. These results indicate that liver perfusate-based biomarker discovery should be limited to liver-specific proteins.

In addition, perfusion conditions cannot entirely mimic true *in-vivo* conditions and may cause stress or ischemia-related injuries, which might affect the relative abundance of certain proteins in the perfusate. Thus, some proteins identified as being differentially expressed in perfusates may result from perfusion, rather than disease-related perturbation. And such false-positive identifications should therefore be ruled out by further validation in plasma or serum.

## Conclusions

In this study, 86 differentially expressed proteins were identified in perfusates from isolated livers metastasized by Walker-256 tumor cells. Functional analysis of the differentially expressed proteins via IPA showed that liver-associated immune function was suppressed by the metastasis of these cells. Proteins associated with liver metastasis were identified in perfusates. Ywhab was among these proteins, and its differential expression in plasma was further validated by Western blotting. The results shown in this study demonstrate the value of utilizing liver perfusates in biomarker discovery for liver diseases.

## Methods

### Establishment of the metastasized model

Walker-256 tumor cells were derived from rat mammary gland carcinosarcomas. These cells have been widely used in cancer studies [7-9]. In the present study, a model of liver metastasis was generated via inoculation of Walker-256 tumor cells into the spleens of male Sprague–Dawley (SD) rats, as described previously [10]. The control rats were subjected to the same surgical procedure as the model rats but were injected with saline solution instead of the tumor cells. This study was carried out in strict accordance with the recommendations in the Guide for the Care and Use of Laboratory Animals of the National Institutes of Health. The protocol was approved by the Institutional Animal Care Use & Welfare Committee of Institute of Basic Medical Sciences (Permit Number: ACUC-A02-2011-046). All surgery was performed under sodium pentobarbital anesthesia, and all efforts were made to minimize suffering.

### Isolated rat liver perfusion

On the 11-13th day after inoculation with tumor cells, the model and control rats were subjected to isolated liver perfusion. The procedure for isolated liver perfusion was similar to that described in a previous publication [3]. The perfusion conditions were in accordance with the standards proposed by Bessems [5]. The perfusate medium was freshly prepared Krebs/Henseleit- bicarbonate buffer (pH 7.4) saturated with a mixture of oxygen and carbon dioxide (95:5). Perfusion flow was mantained at a rate of 3 ml/min.g liver weight. The perfusion temperature was maintained at 37°C.

### Perfusate sample preparation

A total of 10 perfusates were collected, from 5 model rats and 5 normal control rats (Additional file 1: Table S1). The perfusate proteins were extracted via acetone precipitation [3]. The protein extracts were then subjected to quantitation by the Bradford method [28]. Equal amounts of protein derived from five individual rats in the same group (model or control) were mixed together, resulting in two pooled perfusate samples: A model perfusate mixture and a control perfusate mixture.

### Mass spectrometry analysis

The proteins in the two pooled perfusate samples were reduced, alkylated and trypsin-digested as described previously [29]. The tryptic peptides were subsequently desalted via solid-phase extraction (Oasis column, Waters, Inc.) and dried by vacuum evaporation. The dried peptides were resuspended in an aqueous solution containing 0.1% formic acid. In each run, 25 μg of the tryptic peptides was loaded into a 0.32 × 100 mm Polysulfoethyl A (5 μm, 300 Å, PolyLC, Inc.) strong cation exchange (SCX) column and separated into six fractions using the following elution steps: 12.5 mM, 25 mM, 50 mM, 75 mM, 125 mM and 1 M ammonium acetate. Each SCX fraction was loaded in-line onto a peptide trap and then resolved using a 0.1 × 150 mm Magic C18AQ reverse phase column (Michrom Bioresources, Inc.) with the Agilent 1200 HPLC system (Agilent, Inc.). Separation of the peptides was performed at 500 nL/min and was coupled with online analysis via tandem mass spectrometry using an LTQ XL ion trap mass spectrometer (Thermo Finnigan, Inc.) equipped with a Michrom nanospray ionization source (Michrom Bioresources, Inc.). The elution gradient for the reverse phase column ranged from 95% mobile phase A (2% acetonitrile, 0.1% formic acid, 97.9% water) to 40% mobile phase B (10% water, 0.1% formic acid, 89.9% acetonitrile) over 210 minutes. Peptide ions were detected in a survey scan from 400–2000 amu (1 μscan), followed by seven data-dependent MS/MS scans (1 μscan, isolation width of 2 m/z, 35% normalized collision energy, dynamic exclusion for 1 minute). Five technical replicate analyses were performed for each pooled perfusate sample.

All MS/MS spectra were searched against the rat IPI 3.49 protein sequence database (40,131 entries) using SEQUEST algorithm-based Bioworks 3.3.1 (Thermo Finnigan, Inc.). The search parameters were set as follows: precursor mass tolerance, ±2.0 amu; fragment mass tolerance, ±1.0 amu; tryptic cleavage only at lysine or arginine, with up to two missed cleavage sites allowed; and a static modification of +57 amu on cysteine. The search results were further processed with the Trans-Proteomic Pipeline (TPP) (Institute for Systems Biology). The SEQUEST results were validated using PeptideProphet [30], which also calculates the probability of peptide identification. ProteinProphet [31] was applied to assemble the peptides into proteins and calculate the probability of protein identification. The probability of protein identification was calculated based in part on the peptide probability and the SEQUEST Xcorr score [31]. Only proteins identified with a probability >0.9 were considered for further analysis, as this cutoff resulted in a calculated false discovery rate (FDR) of approximately 1%.

### Spectral counting

The spectral count weight (SCW, the proportion of one protein’s spectral count compared to the total spectral count for one MS analysis) was used to estimate the relative abundance of one protein in each sample. Each of the pooled perfusate samples from the model and control rats was subject to five MS analyses, as described above. Ten sets of SCW values were obtained for every protein identified in the ten MS analyses. The SCW value was set to zero when a protein was not identified in an MS analysis. The difference between a protein’s SCW values in the model and control groups was analyzed for statistical significance with Student’s *t*-test using SPSS software (version 11.5). A *p*-value < 0.01 was set as the cut-off for statistical significance.

After quantification, three selection criteria were applied to increase the credibility of protein identification and quantification. First, the proteins identified by a single peptide were excluded, regardless of how many spectra were identified. Second, for proteins showing an elevated relative abundance in the model perfusates, we required that they be identified in all five MS analyses performed in the model perfusates, and for proteins showing an elevated relative abundance in control perfusates, we required that they be identified in all five MS analyses conducted in the control perfusates. Third, we selected proteins showing fold changes > 2.5 as confidently differentially expressed proteins.

### Computational prediction of secretory proteins

SignalP 3.0 [32] (http://www.cbs.dtu.dk/services/SignalP/) was used to predict the N-terminal signal peptides for classically secreted proteins. Two algorithms in SignalP, including neural network algorithm and hidden Markov model, were employed to predict the signal peptides. We considered the proteins that were positively predicted by both algorithms to be secreted.

### Validation of differentially expressed perfusate proteins in plasma by Western blotting

A 20 μl aliquot of plasma collected for validation was first diluted in 1 mL of PBS. Then, the same volume of diluted plasma from every group was loaded onto SDS-PAGE gels and transferred to PVDF membranes (Millipore, 0.22 μm) with a transfer apparatus (Bio-Rad). The membranes were first blocked in a 5% BSA/TBS solution for 1 h at room temperature. All antibodies were purchased from Abcam. The primary antibodies were diluted in a 1% BSA/TBS solution to the ratios indicated in the specific instructions from Abcam, and the membranes were then incubated with the antibodies overnight at 4°C. The membranes were then incubated with the secondary antibodies, diluted 1:10,000 in a 1% BSA/TBS solution, for 2 h at room temperature. The protein signals were detected and quantified with the AlphaEaseFC system.

## Competing interests

The authors declare that they have no competing interests.

## Authors’ contributions

YZ designed the whole experiment, drafted the manuscript and participated in the establishment of the metastasized model, the collection and sample preparation of liver perfusates, proteomic analysis of liver perfusates. ML carried out the immunoassays, participated in the establishment of the metastasized model and bioinformatic analysis of proteomics data. LW helped to establish the metastasized model and collect liver perfusates. LZ offered help in the establishment of the metastasized model and sample preparation of liver perfusates. SH offered help in the bioinformatic analysis. SW offered help in proteomic analysis. SM offered help in the immunoassay. YG conceived of the study, and participated in its design and coordination and helped to draft the manuscript. All authors read and approved the final manuscript.

## Supplementary Material

Additional file 1: Table S1 Lists general characteristics of the perfusates. **Table S2** lists protein numbers and total spectral counts identified in each replicate analysis. **Figure S1** shows serum contamination was low in the perfusates. **Image 1–3** shows macroscopic and microscopic of tumor tubercles. Click here for file

Additional file 2: Table S3 Lists all the differential proteins identified in perfusates. Click here for file
