# Supplementary material for: Differential protein expression in perfusates from metastasized rat livers
Source: Proteome Sci. 2013 Jul 29;11:37. doi: 10.1186/1477-5956-11-37 (PMC3734188; doi:10.1186/1477-5956-11-37)
Supplement: Additional file 1: Table S1 — Lists general characteristics of the perfusates. Table S2 lists protein numbers and total spectral counts identified in each replicate analysis. Figure S1 shows serum contamination was low in the perfusates. Image 1–3 shows macroscopic and microscopic of tumor tubercles. [file 1477-5956-11-37-S1.docx]

**Additional file 1: Table S1.General characteristics of the perfusates.**

|  | **Weight (g)** | **Microscopic**  **metastasis** | **Warm-ischemia time(min)***^a^* | **ALT levels in the perfusates(U)*^b^*** |
| --- | --- | --- | --- | --- |
| **Model Rat**  NO.1  NO.2  NO.3  NO.4  NO.5  **Control Rat**  NO.1  NO.2  NO.3  NO.4  NO.5 | 200  177  172  160  188  280  247  264  217  206 | yes  yes  yes  yes  yes  －  －  －  －  － | 8  5  5  8  5  6  6  6  6  9 | 12  10  7  6  <=5  18  <=5  <=5  <=5  8 |

*a:*The ischemia time was calculated from the initiation of portal vein cannulation to the initiation of perfusion. This may reflect surgery-induced damage to the isolated perfused livers. *b*: The alanine aminotransferase (ALT) levels in the collected perfusates were measured to evaluate liver structural integrity and cytosolic contamination. The difference between the model and control rats was not statistically significant (*p* > 0.05).

**Additional file 1: Table S2. Numbers of proteins and total spectral counts identified in each replicate analysis.**

|  | Protein numbers | Total spectral counts | Overlapping rate |
| --- | --- | --- | --- |
| Model group  Run 1  Run 2  Run 3  Run 4  Run 5  Control group  Run 1  Run 2  Run 3  Run 4  Run 5 | 759  776  742  806  773  620  694  728  657  671 | 17,091  15,750  15,222  17,480  15,568  20,662  19,974  19,555  18,257  17,757 | 75.9%-79.9%  74.2%-80.3% |

**Additional file 1: Figure S1. Serum contamination was low in the perfusates.**

Two micrograms of protein from the perfusate, cytosol and serum mixtures^a^ from the model and control rats were loaded into an SDS-PAGE gel and blotted with an anti-IgG antibody. IgG is a good indicator of serum contamination in perfusates. The small amount of IgG in the perfusates indicated a very low level of serum contamination. M-L: Liver cytosol mixture from the model rats. C-L: Liver cytosol mixture from the control rats. M-P: Perfusate mixture from the model rats. C-P: Perfusate mixture from the control rats. M-S: Serum mixture from the model rats. C-S: Serum mixture from the control rats.

a. The serum mixture was prepared according to our previous work [[1](#_ENREF_1)], by pooling serum samples from five model or control rats.


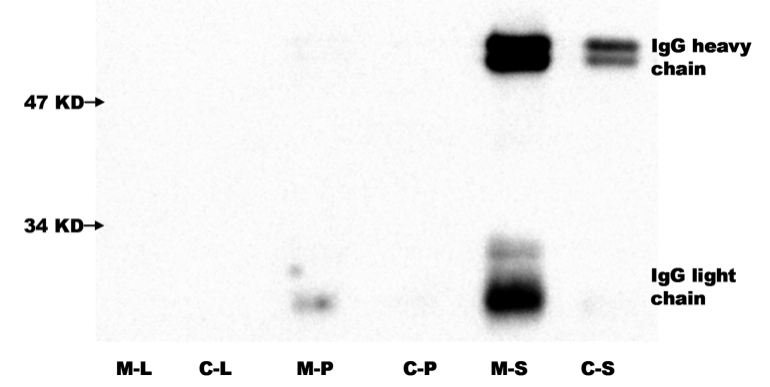


**Additional file 1: Image 1. Macroscopic appearance of tumor tubercles in the spleen.** On the sixth day after inoculation, tumor tubercles were observed in the spleens (arrow).

**
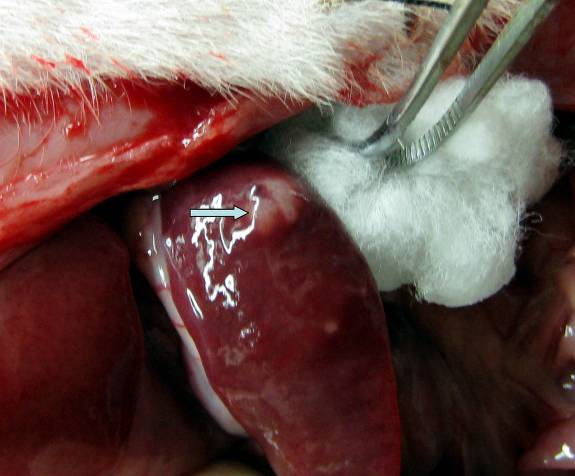
**

**Additional file 1: Image 2.** **Macroscopic appearance of metastasized tubercles in the liver.**

On the ninth day, metastasized tubercles were detected in the livers by visual inspection (arrow).


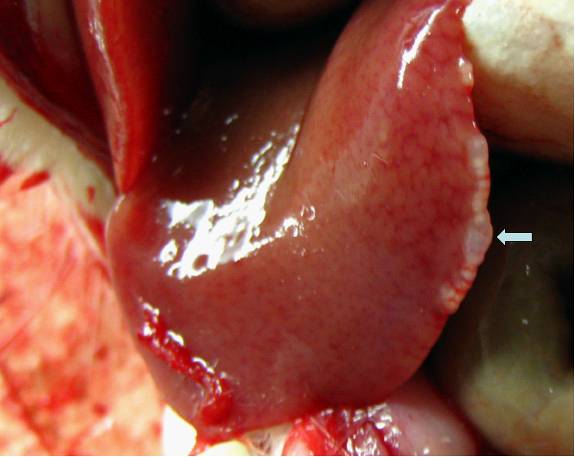


**Additional file 1: Image 3. Microscopic appearance of metastasized tubercles in the liver.**

On the ninth day, metastasized tubercles were detected in the livers by microscopic inspection.


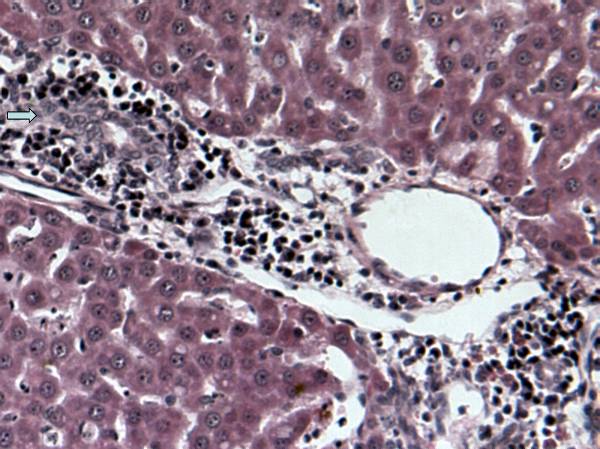


**Reference**

1. Zhang Y, Wang Y, Sun W, Jia L, Ma S, Gao Y: **Strategy for studying the liver secretome on the organ level.** *Journal of proteome research*, **9:**1894-1901.
